# What Can Emergency Medicine Learn From Kinetics: Introducing an Alternative Evaluation and a Universal Criterion Standard for Emergency Department Performance: Erratum

**DOI:** 10.1097/01.md.0000483002.82769.27

**Published:** 2016-04-29

In the article “What Can Emergency Medicine Learn From Kinetics: Introducing an Alternative Evaluation and a Universal Criterion Standard for Emergency Department Performance”,^1^ which appeared in Volume 95, Issue 11 of *Medicine*, Taiwan was not listed as belonging to the Republic of China. The article has since been corrected online.

## References

[1] Pan C-L Chang C-F Chiu C-W What can emergency medicine learn from kinetics: Introducing an alternative evaluation and a universal criterion standard for emergency department performance. *Medicine.* 2016 95:e2972. 26986107 10.1097/MD.0000000000002972 PMC4839888

